# Leading from the frontlines: community-oriented approaches for strengthening vaccine delivery and acceptance

**DOI:** 10.1186/s12919-023-00259-w

**Published:** 2023-06-30

**Authors:** Baldeep K. Dhaliwal, Rajeev Seth, Betty Thankachen, Yawar Qaiyum, Svea Closser, Tyler Best, Anita Shet

**Affiliations:** 1 Department of International Health, Johns Hopkins Bloomberg School of Public Health, Baltimore, MD USA; 2 International Vaccine Access Center, Johns Hopkins Bloomberg School of Public Health, Baltimore, MD USA; 3 Bal Umang Drishya Sanstha, New Delhi, India; 4 Center for Communication Programs, Johns Hopkins Bloomberg School of Public Health, Baltimore, MD USA

**Keywords:** Vaccine confidence, Community-led, Frontline health workers, Behavior change

## Abstract

**Background:**

Although immunization is one of the most successful public health interventions, vaccine hesitancy and the COVID-19 pandemic have strained health systems, contributing to global reductions in immunization coverage. Existing literature suggests that involving community members in vaccine interventions has been beneficial, but efforts to facilitate community ownership to motivate vaccine acceptance have been limited.

**Methods:**

Our research leveraged community-based participatory research to closely involve the community from conception to implementation of an intervention to facilitate vaccine acceptance in Mewat District in Haryana, an area in India with extremely low vaccination coverage. Through the development of a community accountability board, baseline data collection on vaccination barriers and facilitators, and two human-centered design workshops, our team co-created a six-pronged intervention with community leaders and community health workers. This intervention included involving religious leaders in vaccine discussions, creating pamphlets of local vaccine champions for dissemination to parent and child caregivers, creating short videos of local leaders advocating for vaccines, implementing communication training exercises for community health workers, and implementing strategies to strengthen coordination between health workers and supervisors.

**Results:**

Post-intervention data suggested parents and child caregivers had improvements in knowledge of the purpose of vaccines and side effects of vaccines. They noted that the involvement of religious leaders was beneficial, they were more willing to travel to vaccinate their children, and they had fewer non-logistical reasons to refuse vaccination services. Interviews with community leaders and community health workers who were involved in the creation of the intervention suggested that they experienced higher levels of ownership, they were better equipped to address community concerns, and that vaccine misinformation decreased in the post-intervention period.

**Conclusion:**

Through this unique intervention to strengthen vaccine uptake that incorporated the needs, interests, and expertise of local community members, we developed a community-driven approach to strengthen vaccine acceptance in a population with low uptake. This comprehensive approach is essential to amplify local voices, identify local concerns and advocates, and leverage bottom-up strategies to co-design successful interventions to facilitate long-term change.

## Background

Vaccine hesitancy, defined as a delay in acceptance or refusal of vaccines despite the availability of vaccination services, along with structural and systematic pandemic-related disruptions, have taken a toll on vaccine coverage globally [1–4]. Since 2019, the percentage of children who received three doses of the diphtheria, pertussis, and tetanus, vaccine (DPT), a critical marker for immunization coverage, fell to its lowest point in 30 years; 25 million children did not receive a single dose of the DPT vaccine during this period [1]. Importantly, 18 million of these 25 million children live in low-and-middle income countries, with India in particular witnessing one of the largest sustained drops in childhood immunization [4].

Interventions to motivate vaccine acceptance in vulnerable populations through ‘community ownership’ have been limited in India [5]. Community ownership is an approach that strives to empower all members of a community, including community health workers (CHWs), community leaders, parent and child caregivers, and others, to take a leading role in developing strategies and coordinating interventions [6]. With disruptions on both the supply and demand sides of vaccine uptake, particularly in light of the COVID-19 pandemic, it is essential to identify multi-pronged and multi-level strategies to strengthen vaccine coverage.

Community and religious leaders have played critical roles in the success, and failures, of vaccination campaigns globally. In 2003, religious and community leaders slowed the polio eradication campaign for sixteen months over local rumors associated with vaccination efforts [7]. However, the impasse was resolved through extensive engagement and dialogue; these leaders eventually became advocates for polio vaccine campaigns in their communities [8]. Religious leaders have also been credited with building public confidence and credibility in the Polio Eradication Initiative in India and Pakistan by improving coverage in underserved communities, and by countering resistance to polio vaccination [9]. More recently, grassroots community-based advocates were essential to strengthening vaccine uptake during the COVID-19 vaccine rollout [10]. As religious and community leaders have been essential to vaccination efforts globally, it is important to engage leaders early and often to facilitate long-term success of vaccine uptake. Moreover, there is significant evidence that interventions involving CHWs in rural India have resulted in an increase in vaccine coverage rates for children [11]. Many of these interventions have involved CHWs in mobile reminder campaigns, door-to-door-efforts, and social campaigns with mothers [11]. However, prior intervention efforts have led to short-term gains, and suggested that CHWs are neither provided with sufficient resources, such as materials and supervisory support, to sufficiently support vaccination efforts, nor do they receive comprehensive information or training for the range of issues that they face [12–14]. These issues span from local challenges such as low engagement from the community [15], to broader referral-based challenges including limited transportation, poor coordination, and few health facility resources upon transfer [16–20]. It is essential to overcome these barriers to meaningfully empower CHW involvement in community vaccination efforts.

Our research team of Johns Hopkins Bloomberg School of Public Health (JHSPH) and Bal Umang Drishya Sanstha (BUDS) investigators have previously led research in Mewat, a district in the state of Haryana, India that has a population of 1.09 million. Mewat, a large district, is made up of many villages with each of these villages having populations of several thousands of people who have unique barriers to, and hesitancies about, vaccines. Mewat also has a disproportionately high number of unvaccinated children, as only 53.8% of children under two were reported to be fully immunized with Bacillus Calmette-Guérin, measles, and three doses each of the polio and DPT vaccines, based on India’s National Family Health Survey-5 (NFHS-5) data [21]. Our team led previous research in five villages in Mewat, which aimed to understand stakeholder perspectives of vaccination services through focus group discussions. Results indicated that while CHWs were knowledgeable and played a highly influential role in vaccine acceptance and uptake, their role as a bridge between the community and health system was underutilized [22].

Our project, the Community Health Worker-Led Intervention for Vaccine Information and Confidence (CIVIC) Project built on these previous learnings, and aimed to leverage CHWs’ local expertise and strengthen their role in vaccine uptake, thus demonstrating an evidence-based participatory approach to improve community vaccine acceptance.

## Methods

**Fig. 1:**
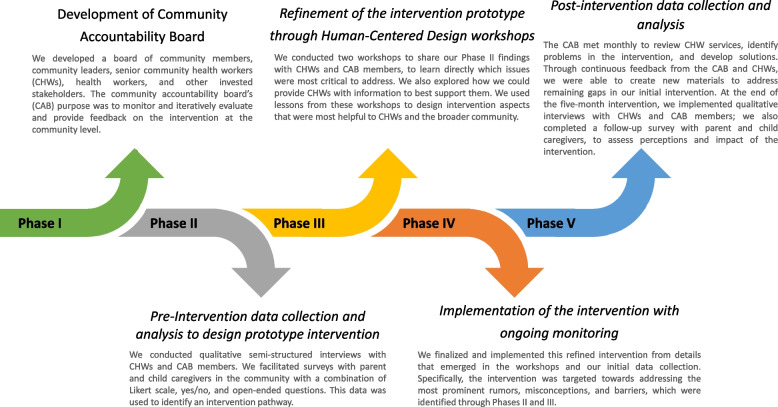
Five-phased approach to developing a community-level intervention

Through the CIVIC project, we facilitated community-based participatory research by closely involving CHWs and community leaders in the design, implementation, and evaluation of a vaccine intervention. Through this five-phased approach, we aimed to achieve a sustainable community-level intervention to facilitate long-term improvements in vaccine acceptance and uptake (Fig. 1).

### Phase I: Development of community accountability board

The BUDS-JHSPH research team initiated Phase I of the CIVIC Project by developing a Community Accountability Board (CAB). In this process influential community members, including senior CHWs, religious leaders, teachers, sarpanches (council leaders), and village elders, were identified and recruited to participate throughout the entirety of the project from design to intervention evaluation. Based on BUDS’ deep knowledge of the community, they first identified individuals who were strongly involved in Mewat, and they individually spoke to each of these members. The BUDS team took notes on their availability, community ties, and involvement in vaccination efforts in Mewat. To further identify members who most aligned with our goals of strengthening vaccine uptake, but were also influential in the community, we engaged in a power-mapping exercise [23]. After identifying community members, the JHSPH-BUDS team requested these individuals to join the CAB. They were asked to assist with providing perspectives on community acceptance of vaccines, be involved in the design of the intervention, and meet on a monthly basis to discuss the progress of the project. Although these monthly meetings were facilitated by the JHSPH-BUDS team, the topics of discussion were driven by the CAB members. The goal of the CAB was to build long term sustainability of the intervention through community ownership.

### Phase II: Pre-Intervention data collection and analysis to design prototype intervention

In Phase II, the JHSPH-BUDS team initiated data collection between January and February 2021 to improve our understanding of the barriers surrounding vaccine acceptance in Mewat. We conducted qualitative in-depth interviews with CHWs and CAB members. These interviews were individually and telephonically facilitated, with each interview lasting between 20 and 60 min. These interviews were led by a member of the BUDS team in Hindi, recorded, and interview notes were taken in English. Ten CHWs and ten CAB member were interviewed in this initial phase of data collection. Interview domains included: current vaccine acceptance in the community, local misinformation or misunderstandings about vaccines, influential community voices, and concerns and hesitations relating to the COVID-19 pandemic and future COVID-19 vaccines. To capture community perspectives, the JHSPH-BUDS team used available lists of parent and child caregivers who have children under five in Ghasera village in Mewat. These lists were used to randomly select parent and child caregivers to participate in telephonic surveys. These survey questions aimed to capture knowledge, attitudes, and practices on vaccination for children through a combination of Likert scale, yes/no, and open-ended questions. The BUDS team completed 30 baseline surveys with parent and child caregivers including mothers, fathers, and grandmothers. Both qualitative and survey data provided baseline information on vaccine acceptance, misinformation on vaccines, community rumors, perceptions of the COVID-19 pandemic and vaccination from CAB members and CHWs, as well as baseline knowledge, attitudes, and practices of parent and child caregivers in the community.

### Phase III: Refinement of the intervention prototype through Human-Centered Design workshops

After this initial understanding of barriers and facilitators, we conducted two human-centered design (HCD) workshops in March 2021. Each of these workshops lasted two hours, they were conducted on an online platform (Zoom), and they were attended by the CHWs and CAB members who were interviewed in Phase II. Prior to conducting the workshops, the BUDS team taught CHWs and CAB members how to download and use the platform functions, including how to chat, use the video and mute functions, and re-join the call in case of any network issues. Through these HCD workshops, we aimed to involve and prioritize the needs of community members at the intervention design phase. In these workshops, the JHSPH-BUDS team provided a preliminary prototype of a multi-pronged intervention, which had been developed based on the data collected in Phase II. Together with the CHWs and CAB members, we jointly refined and designed the nuances of this intervention to be tailored and made most appropriate for the context. CHWs and CAB members led the design of materials that they felt was missing from the current intervention, they shared what was most necessary to learn, and, lastly, they clarified which aspects of our intervention prototype were unnecessary. Through these workshops we aimed to design a comprehensive intervention to motivate and sustain long-term vaccine uptake.

### Phase IV: Implementation of the intervention with ongoing monitoring

Phase IV of the CIVIC Project was initiated after completing our HCD workshops, where the JHSPH-BUDS team finalized and implemented a five-pronged intervention based on components CHWs and CAB members deemed most critical to strengthen vaccine acceptance and uptake in Mewat. Specifically, the intervention was targeted towards addressing the most prominent rumors, misconceptions, and barriers, which were identified in the preliminary data collection. We implemented the intervention refined through workshops conducted in April 2021. During this time, the CAB met monthly to discuss the progress of the intervention, as well as discuss aspects of the intervention which needed to be adjusted to facilitate a larger impact on vaccine uptake. Although these meetings were facilitated and planned by the CIVIC team, the CAB members guided and led the discussions on topics that they felt were most important.

### Phase V: Post-intervention data collection and analysis

Lastly, at the end of these five months, we implemented a rapid evaluation where we conducted ten qualitative interviews with CHWs and nine qualitative interviews with CAB members. We also completed follow-up surveys with parent and child caregivers to assess community perceptions of the intervention. We conducted rapid thematic analysis on the data to assess perceptions on the intervention.

We obtained ethical approval for the study from the Johns Hopkins Bloomberg School of Public Health Institutional Review Board and the Ethics Committee of Bal Umang Drishya Sanstha. Oral consent was collected from all participants prior to engaging in data collection.

## Results

Results from phases of the CIVIC project contributed to the design, implementation, and evaluation of the intervention.

### Phase I: Make-up of community accountability board.

The CAB was made of three women and seven men, who represented a wide range of ages to maximize diversity in feedback. The CAB was made up of one Sarpanch (community council leader), two teachers, one senior CHW, one youth leader, one panchayat (local council representative) member, two Hindu religious leaders, and one local doctor. After we lost one CAB member during the devastating second wave of COVID-19 in India, two new Moulanas (senior Islamic religious leaders) joined the CAB in June 2021. Lastly, members of the CAB had a diverse amount of expertise in their respective positions, with years of experience ranging from five to twenty-seven years.

### Phase II: Pre-Intervention data collection and analysis to design prototype intervention

Our team completed a preliminary synthesis of initial data from CHWs, CAB members, and parent and child caregivers. We used notes and recordings from interviews to rapidly transform our research data into meaningful and actionable insights by qualitatively (1) documenting what stood out in conversation; (2) organizing insights into categories; (3) organizing categories into themes; and (4) documenting possible solutions.

Our analysis of pre-intervention data suggested that CHWs, CAB members, and parent and child caregivers perceived that there were communication barriers associated with vaccination activities. Communication barriers ranged from senior CHWs not providing frontline CHWs with sufficient knowledge to communicate with parent and child caregivers, and parent and child caregivers feeling that their concerns were being neglected in the push to rapidly meet vaccination targets set by district and state-level health officials. Religious leaders were commonly identified as a key solution to overcoming communication barriers, as parent and child caregivers expressed that they had deep trust in religious leaders. Moreover, CAB members and CHWs felt religious leaders’ involvement in caregiver outreach would be beneficial to community uptake of vaccines. Results also suggested that gaps in CHW training limited the amount of information that CHWs were able to share with parent and child caregivers, which further impacted willingness to accept vaccines. Lastly, COVID-19 was commonly seen as a problem that was not impacting rural populations like Mewat and was instead plaguing larger cities like Delhi. Members of the CAB and CHWs expressed that parent and child caregivers may be less likely to accept COVID-19 vaccines because of this perception. Key findings are detailed in Table 1.

**Table 1:** Qualitative Baseline Data Key Findings (January – February 2021)

**Parent and child caregivers** *Open-Ended Survey Data Findings*	**CHWs** *Qualitative In-Depth Interviews*	**CAB Members ** and ** CHWs** *Qualitative In-Depth Interviews*
Felt that CHWs provided valuable information regarding vaccines	Did not feel that they had adequate support from supervisors and health officials. They also felt that, at times, they had training gaps which did not allow them to fully answer parent and child caregivers’ vaccination concerns	Identified communication barriers which led to a lack of coordination on vaccine communication
There were perceptions that CHWs did not properly listen to or address concerns at times	CHWs did not fully recognize their role in vaccine acceptance in the community	Felt having support from religious leaders would be impactful in reaching out to community members
Their largest concern around vaccines centered around side effects	Felt that senior CHWs (Auxiliary Nurse Midwives) were not able to provide CHWs with enough time to inform parent and child caregivers about vaccination camps early. This lack of advance warning left parent and child caregivers feeling that they didn’t have enough time to make a decision about vaccines, nor did they have enough time to get questions answered by CHWs or senior CHWs	
The COVID-19 pandemic, at the time of the interviews, was not perceived as a problem that was impacting rural populations like Mewat		Believed community would not be as willing to accept COVID-19 vaccines, as COVID-19 was seen as a ‘city problem’
Religious leaders were perceived to be the most trusted sources of information in the community regarding vaccination		

*CHW* Community Health Worker, *CAB* Community Accountability Board

We used this primary data collection to design an initial prototype of a six-pronged intervention, which would later be refined through HCD workshops (Table 2).

**Table 2:** Initial intervention prototype

Intervention Component (Prototype)	Description of Intervention (Prototype)
Strengthen Communication	Data suggested that providing specific communication training would be beneficial for CHWs. Further data suggested that providing CHWs with training booklets would assist them with speaking to parent and child caregivers in less technical terms
Improve Coordination between CHWs and Senior CHWs	Data suggested that if senior CHWs were able to share select information with CHWs one week in advance of a vaccination campaign, this might encourage parent and child caregivers to accept vaccines. Data suggested it would be effective to share information in advance with parent and child caregivers on (1) the timing of vaccines; (2) which vaccines are available; (3) side effects; (4) how to anticipate and treat side effects with simple home treatments
Direct Vaccine Material for Parents	Providing information to caregivers in easy-to-read pamphlets was suggested to alleviate vaccine concerns
Involving Religious Leaders	As religious leaders are influential in this community, our data suggested that involving them in vaccination campaigns was essential to improving vaccine uptake
Policy Level Changes	Data suggested that many parent and child caregivers were unable to take their children for vaccines during the harvest season, as this was associated with loss of income. Further, the Pentavalent vaccine was specifically associated with parent and child caregiver frustration, as this led to many side effects and was a high contributor to hesitancy. Making policy recommendations and changes was one way to alleviate these issues
COVID-19 Communication Campaign	As the community did not perceive COVID-19 to be an issue at the time of primary data collection, data suggested that vaccine uptake would be difficult and slow unless communication about the COVID-19 vaccine began early

This prototype of an intervention was presented to CHWs and CAB members in our HCD workshops (Phase III).

### Phase III: Refinement of the intervention prototype through Human-Centered Design workshops

In these workshops CHWs and CAB members provided their expertise on which aspects of the prototype would be effective, how interventions could be implemented, which aspects would be too challenging to complete, and which aspects could be longer-term goals. In the initial HCD workshop with CHWs, they shared that additional booklets were not necessary; as booklets have already been provided to CHWs, they didn’t feel that having an additional booklet would translate to vaccine uptake in the community. CHWs also felt that any direct vaccine information for parent and child caregivers would need to be simple or pictorial, as there is poor literacy among women in Mewat. Providing pamphlets with too much text, was seen as an ineffective intervention. During the HCD workshop with CAB members, one senior CHW, who also served as a CAB member, shared that she would be unable to provide the level of detail requested for vaccination campaigns a week in advance. However, she agreed that this idea was essential to strengthen vaccine confidence among parent and child caregivers; she suggested that she would work with other senior CHWs to alert CHWs to vaccine campaigns via WhatsApp in advance. Advocating for policy-level changes was also something that CHWs and CAB members felt would be unfeasible in the duration of this project. Lastly, coordinating a large-scale COVID-19 vaccine communication campaign, so close to vaccine introduction, was also seen as a difficult task. CHWs felt that implementing a large-scale communication campaign would be challenging, particularly considering other COVID-19 responsibilities. After discussions with the CHWs and CAB members, we decided a feasible alternative would be to provide CHWs and CAB members with communication information on COVID-19 vaccinations, so they could instead disseminate this information to community members through their conversations.

### Phase IV: Implementation of the intervention with ongoing monitoring

After the HCD workshops and feedback from CHWs and CAB members, we refined and created an updated five-pronged intervention to strengthen vaccine uptake in Mewat (Table 3).

**Table 3:** Co-created refined five-pronged intervention

**Intervention Component:** *Refined through HCD Workshops*	**Description of Intervention** *Designed by CHWs and CAB members*
Involvement of Religious Leaders (Maulanas)	Religious leaders joined the CAB for the final months of the project, where they were more deeply involved with sharing strategies to support vaccine uptake. Religious leaders also participated in advocacy videos which could be both disseminated via WhatsApp and displayed when CHWs spoke to parent and child caregivers in the community
Pamphlets of Vaccine Champions	A total of three pamphlets were created. Two pamphlets were designed to target routine vaccinations, as well as parent and child caregivers’ fear of side effects. The third pamphlet displayed a village elder, who was previously a CHW in Mewat, who had accepted COVID-19 vaccines. This pamphlet was to create demand for COVID-19 vaccines in the community
Videos of Vaccine Champions	Our team initially created two videos with two respected members of the CAB, a social worker and teacher. *After feedback from the CAB, we created an additional video of a prominent religious leader (maulana).* These advocates discussed the importance of vaccination, the need to complete immunization in a timely manner, and the negative long-term impacts of not vaccinating a child
Training and Exercises	We developed and led trainings on interpersonal communication and effective communication around COVID-19 vaccines, including on side effects. Our team also developed and led two exercises focused on reflective listening and journey mapping, which allowed CHWs to practice these new skills
Improved Coordination between CHWs and Supervisors	We coordinated with ANM workers, both in and out of the CAB, to develop a more established and consistent visit schedule for vaccine delivery in Mewat

The first prong of our intervention targeted the involvement of religious leaders in vaccination efforts. Our team worked closely with religious leaders to address their vaccination concerns, encourage their involvement in CAB meetings, and motivate them to participate in vaccine advocacy. Once we had thoroughly addressed their concerns and gained their trust, they agreed to participate in the remainder of our intervention and serve as vaccine champions.

The second aspect of our intervention centered around the design of largely pictorial pamphlets which could be used by CHWs to initiate and facilitate easy discussions with parent and child caregivers. Pamphlets addressed the most prominent issues perceived in the community, and they used language that was most appropriate for the parent and child caregivers in this setting. These pamphlets displayed well-known and influential members of the community who had received vaccines themselves, or who had vaccinated their children.

Our team also created three videos of respected community members who were willing to advocate for vaccines, as this would provide CHWs with additional materials that would facilitate their discussions with community members. The videos included both religious leaders and members of the CAB. Videos were designed to be short, easy-to-use in conversations with parent and child caregivers, and easy to disseminate on WhatsApp among parent and child caregivers. The videos also filled the literacy gap highlighted in our HCD workshops, as they were beneficial for individuals who were unable to read the words on the pamphlets.

The fourth aspect of our intervention involved addressing the CHWs’ communication and knowledge gaps that were identified in earlier phases of our project. These specific knowledge and communication gaps were a result of limited CHW training, in these particular areas. Our trainings were designed to distill complex topics, which CHWs had not previously had exposure to, into easy-to-digest material. We further ensured walkthrough exercises provided CHWs with opportunities to practice these new skills.

Lastly, we worked with senior CHWs to address parent and child caregiver and CHW concerns that there was not sufficient time to ask or answer questions about vaccines on vaccination days. As providing parents with time to ask any questions and giving CHWs time to build confidence in vaccines may improve uptake, senior CHWs developed and initiated a more consistent schedule for vaccination services in Mewat. They also provided advance notice to CHWs for vaccination days so CHWs could, in turn, speak with parent and child caregivers early.

### Phase V: Post-intervention data collection and analysis

During monthly CAB meetings, we made the following additions to strengthen our intervention: (1) we included Maulanas in the CAB, as opposed to requesting them to serve as advocates; (2) we created an additional video of a maulana advocating for vaccines; (3) CAB members began to assist CHWs with reaching out to hesitant parent and child caregivers during CHWs door-to-door visits; and (4) CAB members began to attend vaccination clinics to instill vaccine confidence in parent and child caregivers.

After completing the intervention, we found that parent and child caregivers had improvements in their knowledge of the purpose of vaccination and on side effects of vaccines. Second, parent and child caregivers also noted that the involvement of community and religious leaders in vaccination efforts was welcome. Lastly, parent and child caregivers expressed that they were more willing to travel to vaccinate their children, and they had fewer non-logistical reasons to refuse vaccination services. Interviews with CHWs and CAB members stated that they felt greater ownership over vaccination in their community, and CHWs felt more prepared to address community concerns with routine and COVID-19 vaccine misinformation. However, CHWs and CAB members noted that additional gaps remained. Tables 4 and 5 inlude the results of a rapid thematic analysis of these topics.

**Table 4:** Phase V key findings from CHWs

Finding	Detail	Quote
**Physical items (videos and pamphlets) were most helpful to CHWs** **in their communication with parent and child caregivers**	CHWs shared that when they were attempting to address caregiver concerns, they were most convinced by seeing evidence of other members of their community who benefited from vaccination or from others advocating for vaccination	*“Now I don’t have [a] fear of going alone since I have these [pamphlets], I feel like a helper is coming along with me.”*
**Communication training was a helpful method of training for CHWs**	CHWs described that they previously had not been provided with effective communication training. When speaking with hesitant parent and child caregivers, CHWs previously found it difficult to understand how to speak effectively and in a way that parent and child caregivers responded well to	“*Communicating about the vaccination has always been a challenge for us, but now this problem has been lightened through this intervention”*
**Strengthened connections with community leaders** **facilitated improvements in community-level discussions**	CHWs shared that their links with community leaders were improved, and they felt that they had additional support with parent and child caregivers who were hesitant about vaccination	“*Earlier only we were working all alone. Now we have helping hands”*
**CHWs felt that community members were more willing to accept vaccines after the implementation of this intervention**	CHWs shared that there were improvements in both routine immunization services and COVID-19 vaccination. They credited this improvement to some aspects of the intervention including communication, coordination, and strengthened connections	“*Earlier people refused to take vaccines. Now people are voluntarily coming to the session and taking vaccines.”*
**CHWs felt that the intervention allowed them to develop new technological and general communication skills**	CHWs shared that the intervention allowed them to feel more confident in themselves to use online platforms, discuss issues with parent and child caregivers beyond vaccination, and improve relationships with parent and child caregivers by leveraging support from the CAB	“*I learnt to be patient with parent and child caregivers, talk peacefully, give them time to think and react, and communicate to them about vaccines properly.”* *“The online exposure was also a wonderful experience sitting in home itself meeting people [from] far [away], [helped with] learning.”*
**CHWs felt increased o**wnership and** empowerment** over vaccine uptake in** Mewat**	CHWs shared that they feel more empowered to apply skills to facilitate change. They also shared that they feel more prepared to help with larger health issues	*“[When] training is provided to us for developing our skills, we learn and implement it in our field. We are helping to bring [in gradual] change.”* *“We would like to [have] more materials later also, as through [these strategies] we can explain the caregiver in a better way. Pamphlets regarding pregnant women, nutrition, vaccines. Religious leaders’ messages can also be added.”*

**Table 5:** Phase V key findings from CAB members

Finding	Detail	Quote
**Videos were helpful in communicating with parent and child caregivers**	CAB members shared that when they were attempting to address caregiver concerns, they were most convinced by seeing evidence of others who they trust advocating for vaccination. The religious leaders’ videos were particularly impactful	“*Video circulated through WhatsApp has inspired the community members. Our team members motivated and worked together.”*
**Religious leaders play a very important role in improving vaccination in Mewat**	CAB members shared that before the religious leaders became fully involved, the intervention had limited impact. Once they fully engaged with the project through meetings and videos, parent and child caregivers began to be more accepting of vaccination	“*The religious leader video has been a very useful aspect of the intervention. During talks, I have shown these videos while communicating about vaccination then [community members] suddenly made a decision [to accept] vaccine[s].”*
**The community-driven, diverse, approach of the intervention was a useful approach**	Members of the CAB felt that the most successful impacts of the intervention were due to the local community-driven approach. Having trusted members of the community helped ensure the success of the intervention	“*The pamphlet of the local champion, especially of Covid vaccination, was a dynamic one. The candidate chosen for this is a reputed elderly woman. The community member accepted her face and paid attention to it.”*
CAB members felt that community** members were more willing to accept vaccines after the implementation of this intervention**	Members of the CAB shared that there were improvements in both routine immunization services and COVID-19 vaccination	*“Earlier the COVID vaccination and information in Ghasera was zero in all the sessions but now it has risen to 100 or 150 or 200 per session daily. Now Vaccine rumor has diminished. The present vaccination (Covid) trend here has bounced greatly.”*
**CAB members felt that there is a need to address larger systemic problems in Mewat, which contribute to low vaccine uptake**	The CAB felt that progress had been made in terms of vaccination, but many felt that this was not addressing the root of the problem. They felt that further meeting to discuss issues beyond vaccination would work towards improving more broad issues in Mewat, such as limited schooling for girls, and limited discussions of vaccines in India	“*Girls over here are less educated (of 4*^*th*^* – 6*^*th*^* grade) or uneducated. If we focus on educating these girls then all these situations related to vaccination can be managed.”*

## Discussion

The CIVIC project facilitated engagement and collaboration between CHWs, community leaders, and members of the community. By building on community-based participatory research strategies, we were able to create networks to support more discussions about vaccination as well as facilitate parent and child caregiver self-efficacy to access and accept vaccines.

To facilitate community-driven solutions in future work, we found that it is essential to acknowledge, articulate, and amplify the values that members of the community feel are important. By combining new approaches to strengthen vaccine uptake, specifically by bringing the needs, interests, and expertise of Mewat community members to the heart of all approaches, we created a scalable methodology to address vaccination beliefs and behaviors in a population with low vaccine uptake. To create long-lasting solutions to vaccine barriers, researchers should begin by engaging deeply with the community to identify barriers and facilitators to vaccination. Researchers should then partner with the community leaders, as they are the local experts, to prototype and refine an intervention tailored to the vaccination needs of the community. By investing the time and resources to allow the community to craft viable solutions, we can work towards creating solutions that will be sustainable in the long-term. Lastly, it is essential to support community leadership, such as a CAB, to develop and sustain a successful health intervention beyond the period of the project. We believe that this approach is critical to sustainable, equitable, long-term change, as it allows for members of the community to have the decision-making authority, information, and time to craft solutions to best benefit members of their own community.

This project was limited by several factors. There is a possibility of volunteer bias, as participants who have extreme positive feelings about vaccination services might have been more willing to participate in the CAB. If we had been able to include participants who were less positive about vaccines, we may have been able to design a broader range of interventions. Another limitation of our study is the possible confirmation bias during the HCD workshops when CHWs and CAB members were asked to share their perspectives on the preliminary prototype of the intervention. Although attendees did share which aspects they did not feel were needed, it is possible that when presenting our preliminary intervention, attendees did not feel comfortable disagreeing more broadly. Additionally, we did not rely on our survey data to assess the direct impact of the intervention, as our sample size was convenience-based for feasibility, and it was not powered to show an impact. However, the qualitative data that we collected effectively assessed perceptions of the intervention from multiple community perspectives. Lastly, the COVID-19 pandemic impacted multiple aspects of our project. CHWs had an increased workload in terms of COVID-19 response activities, lessening their ability to engage with the CIVIC project, and we were also unable to safely conduct in-person events which may have facilitated additional vaccine uptake.

## Conclusion

Vaccination needs, hesitations, and barriers, are not homogeneous, and in a country as large as India, they can vary village to village, or at an even more granular level. This variation is shaped by the experiences, history, context, and broader health needs in communities, which requires solutions to be hyper-localized to the setting. Leveraging ‘top–down’ approaches in the context of intervention design and implementation, as has been done repeatedly, creates the risk of leaving community voices behind. Policymakers, researchers, and local organizations should adopt local approaches to provide communities both a voice and resources to turn their needs and ideas into action. This comprehensive approach can amplify local voices, identify local concerns and advocates, and leverage ‘bottom-up strategies’ to co-design successful interventions to facilitate long-term change.

## Data Availability

Data sharing is not applicable to this article as no datasets were included in the manuscript and included analysis. The interviews analyzed and meeting minutes are available from the corresponding author upon reasonable request.
